# Underweight body mass index is a risk factor of mortality in outpatients with nocturia in Japan

**DOI:** 10.1186/s13104-015-1456-6

**Published:** 2015-09-29

**Authors:** Hiromitsu Negoro, Yoshio Sugino, Koji Nishizawa, Takeshi Soda, Yosuke Shimizu, Kenichi Yoshimura, Osamu Ogawa, Koji Yoshimura

**Affiliations:** Department of Urology, Kyoto University Graduate School of Medicine, Kyoto, Japan; Department of Urology, Kurashiki Central Hospital, Okayama, Japan; Department of Clinical Trial Design and Management, Translational Research Center, Kyoto University Hospital, Kyoto, Japan; Department of Urology, Kobe City Medical Center General Hospital, Kobe, Japan; Department of Urology, Shiga Medical Center for Adults, Shiga, Japan; Department of Urology, Kitano Hospital, Osaka, Japan; Department of Urology, West Kobe Medical Center, Kobe, Japan; Innovative Clinical Research Center, Kanazawa University, Kanazawa, Japan; Department of Urology, Shizuoka General Hospital, 4-27-1 Kita Ando, Shizuoka Aoi-ku, Shizuoka, Japan

**Keywords:** Body mass index, Mortality, Nocturia, Risk factors

## Abstract

**Background:**

Although nocturia has been reported to increase mortality in elderly individuals, the particular risk factors that are associated with this event are unclear. Therefore, we evaluated risk factors for death in outpatients with nocturia.

**Methods:**

Between October 2002 and December 2009, 250 consecutive patients with nocturia were enrolled in two general hospitals in Japan. Among them, 193 patients were able to be followed for at least 1 year and up to 9 years (median 4.8 years) if the patients did not die. Mortality rates and risk factors were evaluated in the nocturic outpatients.

**Results:**

Two- and 5-year survival of the nocturic outpatients was 94.6 % [95 % confidence interval (CI) = 92.2–97.1] and 82.6 % (95 % CI = 75.4–87.8), respectively. Higher Charlson Comorbidity Score, lower body mass index (BMI) and lower Physical Component Summary of Short Form-36 item scores were significantly correlated with mortality (*p* < 0.0001, *p* < 0.005 and *p* < 0.05, respectively) in multivariate analysis. The International Prostate Symptom Score, Pittsburgh Sleep Quality Index, Mental or Role/Social Component Summary of Short Form-36 item scores and Nocturnal Polyuria index were not significantly correlated with mortality. The mortality rate was significantly higher in subjects with an underweight BMI (<18.50) compared with a normal range (18.50–24.99) or overweight (≥25.00) BMI [*p* < 0.00005, hazard ratio (HR) = 5.84, 95 % CI = 2.03–16.8; *p* < 0.0005, HR = 5.92, 95 % CI = 1.94–18.0].

**Conclusions:**

Additional attention is required for nocturic outpatients with not only a high Charlson Comorbidity Score but also an underweight BMI because of their high mortality. Large prospective studies are warranted to validate this finding and extend more.

**Electronic supplementary material:**

The online version of this article (doi:10.1186/s13104-015-1456-6) contains supplementary material, which is available to authorized users.

## Background

Nocturia is a common lower urinary tract symptom (LUTS), which is associated with impaired quality of life (QOL), particularly when two or more episodes occur per night [1, 2]. Several epidemiological studies have reported increased mortality in people with nocturia [3–6]. According to the Third National Health and Nutrition Examination Survey, which included more than 15,000 people, there is a significant trend of increased mortality risk with increased number of nightly voiding episodes in men and women [5]. Bursztyn et al. suggested that nocturia was a significant independent predictor of mortality among 70-year-old patients with coronary heart diseases [4]. These studies have provided important information regarding the effect of nocturia on mortality. However, the risk factors of morality in nocturic patients remain unclear, and a direct relationship between nocturia and mortality has not been clearly shown yet [7].

Because the cause of nocturia is multifactorial, including prostatic hyperplasia, age, psychological aspects, and medical problems, such as hypertension, diabetes, stroke, and obesity, the cause-and-effect associations are not always obvious [7]. These multifactorial and unclear aspects can also be applied to the cause of death in nocturic patients. However, nocturic patients might have a common risk factor in death. Therefore, we retrospectively reviewed the survival rate of outpatients who attended large general hospitals with the chief complaint of nocturia, and evaluated risk factors for death using multivariate statistics.

## Methods

A total of 250 patients who attended the urology section of two general hospitals (Kurashiki Central Hospital and Kyoto University Hospital) with the chief complaint of nocturia (two or more episodes) between October 2002 and December 2009 were enrolled in this study with written informed consent. We reviewed the patients’ records to determine their survival and causes of death. When insufficient data were achieved from the records, we used postal mail and telephone calls. The study conformed to the provisions of the Declaration of Helsinki and was approved by Kyoto University Graduate School and the Faculty of Medicine Ethics Committee (E-1037).

The follow-up period was at least 1 year and up to 9 years (median 5 years) if the patients did not die. We also evaluated age at the first visit, sex, body mass index (BMI), Charlson Comorbidity Score [8], and scores on several questionnaires administered at the first visit, to analyze correlates of mortality (Additional file 1). The number of nocturnal voids was evaluated using the seventh question of the International Prostate Symptom Score (IPSS). The questionnaires used in this study were the IPSS for assessment of general LUTS [9], the Medical Outcome Study Short Form-36 item (SF-36) for health-related QOL (HRQOL) [10, 11] and the Pittsburgh Sleep Quality Index for sleep status [12]. The Japanese versions of these questionnaires have been previously validated [13–15] The Nocturnal Polyuria index (NPi), determined as nocturnal urine volume divided by total urine volume [1], was calculated from the bladder diary for at least 2 days. Prospectively collected data from these questionnaires were analyzed retrospectively in this study. Survival curves were obtained by the Kaplan–Meier method. We used Cox’s proportional hazards model to assess risk factors for death. The assumption of proportional hazards was checked by testing for time-dependent hazards. Risk factors included age at first visit, sex (categorized), BMI, IPSS score 7, IPSS total score, IPSS QOL, SF-36 PCS, SF-36 MCS, SF-36 RCS, PSQI, NPi and Charlson Comorbidity Score. We performed forward stepwise regression analysis to determine the association between several factors and a death in nocturic patients. Interaction between BMI and other factors were also examined. The SPSS ver.11.0.1J software (SPSS Inc., Chicago, IL, USA) was used statistical analyses. Survival curves were obtained by the Kaplan–Meier method with BMI categorized into three groups according to the WHO criteria (underweight <18.50; normal range 18.50–24.99; overweight ≥25.0).

## Results

We were able to follow 193 (77.2 %) patients up to 110 months (median 58.2 months) and 40 (20.1 %) patients died. The baseline characteristics of the study population are shown in Table 1 and in Additional file 1. Two- and 5-year survival of the nocturic outpatients was 94.6 % (95 % CI = 92.2–97.1) and 82.6 % (95 % CI = 75.4–87.8), respectively. Kaplan–Meier estimates of all-cause mortality in outpatients with nocturia are shown in Additional file 1. The causes of death of the nocturic patients were malignancy (n = 16, 40.0 %), respiratory infection (n = 5, 12.5 %), hematologic disease (n = 3, 7.5 %), cardiovascular disease (n = 2, 5.0 %), diabetes mellitus (n = 2, 5.0 %) and others (n = 12, 30.0 %; including traffic accidents, rheumatism, pneumothorax, spinocerebellar degeneration, suicide, senility, spinal infraction, and unknown).

**Table 1:** Clinical characteristics of the study population

Characteristics	Total
No. patients	193
Age, median (IQR) (years)	73 (11)
Female, no. (%)	37 (19.2)
BMI, median (IQR)	22.5 (3.4)
IPSS score 7, median (IQR)	4 (1)
IPSS total score, median (IQR)	15 (9)
IPSS QOL, median (IQR)	4 (1)
SF-36 PCS, median (IQR)	46.0 (14.6)
SF-36 MCS, median (IQR)	49.8 (12.9)
SF-36 RCS, median (IQR)	47.0 (23.0)
PSQI, median (IQR)	8 (6.8)
NPi, median (IQR)	0.43 (0.32)
Charlson Comorbidity Score	1 (2)
Follow up (years)	4.85 (4.99)

*IQR* interquartile range, *BMI* body mass index, *IPSS* International Prostate Symptom Score, *SF-36* Medical Outcome Study Short Form-36 item for health-related QOL, *PCS* Physical Component Summary, *MCS* Mental Component Summary, *RCS* Role Component Summary, *PSQI* Pittsburgh Sleep Quality Index, *NPi* Nocturnal Polyuria index

Univariate analysis showed that age at the first visit (*p* ≤ 0.001), low BMI (*p* ≤ 0.0001), low SF-36 Physical Component Summary score (*p* ≤ 0.01) and Charlson Comorbidity Score (*p* ≤ 0.0001) were significant correlates of mortality, while frequency of nocturia shown by IPSS score 7 or relative nighttime urine production indicated by NPi was not (Table 2). In multivariate analysis, low BMI, low SF-36 Physical Component Summary score and high Charlson Comorbidity Score were significantly correlated with mortality [*p* < 0.005, hazard ratio (HR) = 0.83, 95 % confidence interval (CI) = 0.74–0.94; *p* < 0.05, HR = 0.97, 95 % CI = 0.95–1.00; *p* < 0.0001, HR = 1.44, 95 % CI = 1.21–1.71, respectively; no significant interaction was observed between BMI and Charlson Comorbidity Score or SF-36 PCS] (Table 2).

**Table 2:** Cox regression analyses predicting the death in nocturia patients

	Univariate analysis	
	OR (95 % CI)	*P* value*
Age at first visit	1.09 (1.04–1.14)	<0.001
Female	0.53 (0.21–1.35)	0.18
BMI	0.81 (0.73–0.90)	<0.0001
IPSS score 7	1.17 (0.83–1.64)	0.38
IPSS total score	1.01 (0.96–1.01)	0.78
IPSS QOL	1.17 (0.88–1.56)	0.29
SF-36 PCS	0.97 (0.94–0.99)	<0.01
SF-36 MCS	0.99 (0.96–1.03)	0.54
SF-36 RCS	0.99 (0.96–1.01)	0.3
PSQI	1.00 (0.91–1.09)	0.93
NPi	0.59 (0.06–5.60)	0.64
Charlson Comorbidity Score	1.42 (1.24–1.63)	<0.0001

	Multivariate analysis	
	OR (95 % CI)	*P* value*
Age at first visit	1.05 (0.98–1.11)	0.15
BMI	0.83 (0.74–0.94)	<0.005
SF-36 PCS	0.97 (0.95–1.00)	<0.05
Charlson Comorbidity Score	1.44 (1.21–1.71)	<0.0001

**Fig. 1:**
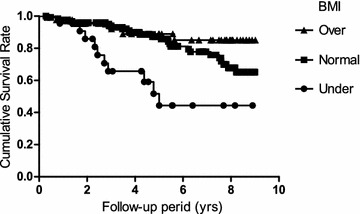
Kaplan–Meier survival curves of the three BMI groups. Under <18.5, normal 18.5–24.99, over ≥25.0

Patients were divided into three groups according to BMI of WHO criteria (underweight <18.50, normal range 18.50–24.99, overweight ≥25.0) (Table 3). Kaplan–Meier curves of the three BMI groups are shown in Fig. 1. The mortality rate was significantly higher in subjects with underweight BMI compared with those with normal range or overweight (*p* < 0.00005, HR = 5.84, 95 % CI = 2.03–16.8; *p* < 0.0005, HR = 5.92, 95 % CI = 1.94–18.0).

**Table 3:** Clinical characteristics of the patients according to the BMI

Characteristics	BMI			*P* value*
	<18.50	18.50–24.99	≥25.00	
No. patients	22	128	43	
Age (years), median (IQR)	75 (10)	73 (11)	70 (9)	<0.05
Female, no. (%)	7 (31.8)	23 (18.0)	7 (16.3)	0.27
BMI, median (IQR)	17.4 (1.5)	21.9 (3.1)	27.1 (3.4)	NA
IPSS score 7, median (IQR)	4 (1.3)	4 (1.0)	4 (1)	0.54
IPSS total score, median (IQR)	11 (13.8)	15 (9)	15 (10)	0.74
IPSS QOL, median (IQR)	5 (1)	5 (1)	4 (1.3)	0.21
SF-36 PCS, median (IQR)	40.3 (25.6)	46.1 (14.4)	47.0 (11.6)	0.74
SF-36 MCS, median (IQR)	48.0 (13.4)	50.2 (14.1)	49.5 (12.5)	0.46
SF-36 RCS, median (IQR)	38.9 (30.5)	47.1 (23.3)	47.1 (23.3)	0.3
PSQI, median (IQR)	9 (6)	8 (7)	7.5 (7)	0.81
NPi, median (IQR)	0.46 (0.37)	0.43 (0.32)	0.43 (0.35)	0.59
Charlson Comorbidity Score	1 (2)	1 (2)	0 (2)	0.36
Follow up (years), median (IQR)	4.27 (3.25)	4.73 (4.91)	5.65 (6.07)	0.21

## Discussion

In this study, we showed that underweight BMI, as well as lower Physical Component Summary of Short Form-36 item scores and high Charlson Comorbidity Score, was a risk factor of mortality of nocturic outpatients in two general hospitals in Japan (Kurashiki Central Hospital and Kyoto University Hospital).

Of note, high BMI is suggested as a risk factor for nocturia [16]. Therefore, a higher BMI population tends to be more nocturic than others, similar to metabolic syndrome. However, whether BMI is related to mortality of nocturic patients is unknown. BMI did not have any significant interaction with Charlson Comorbidity Score or Physical Component Summary of Short Form-36 item, indicating that participants did not tend have low BMI simply because they were ill. It has recently been reported that BMI is inversely related to all-cause mortality in elderly people [17, 18]. A large study conducted in Japan showed that a low BMI in elderly people was associated with an increased risk of all-cause mortality, and the results were essentially unchanged, even when the analyses were conducted in those who did not have cancer, cardiovascular disease, or stroke [17]. Moreover, higher BMI patients had improved survival rate in decompensated congestive heart failure [19] and in chronic heart failure [20]. This paradoxical phenomenon was extended to the general population in a systemic review and meta-analysis [21]. The exact reasons are not clear, but explanations for this paradox are that lean mass acts as a nutritional preserve and thin older patients may have less immune response [22]. These factors may apply to nocturic elderly people.

In the present study, the cause of death largely depended on the comorbidities. This is considered to be because subjects were outpatients with nocturia from two relatively large general hospitals, although the survival rate of the nocturic patients in the present study was not lower than that of the Japanese population calculated using age- and sex-specific population estimates released by the Japanese Ministry of Internal Affairs and Communications (using http://www.e-stat.go.jp/SG1/estat/XlsdlE.do?sinfid=000000090264; data not shown). Therefore, we re-analyzed particular groups selected with Charlson Comorbidity Score between 0 and 2 (n = 166) in the same manner (Additional file 1). The result showed a clearer impact of BMI solely on the mortality, with hazard ratio of 0.73 (95 % CI 0.62-0.88) in multivariate analysis. The Charlson Comorbidity Score was not significantly related to the mortality in this particular group, indicating that multiple comorbidities rather than the individual factor could drive the event. In addition, the overweight BMI group had a significantly better survival rate than the normal BMI group, with hazard ratio of 3.00 (*p* < 0.05, 95 % CI = 1.01–8.94). This result suggests that although obesity is reported to be a risk factor of nocturia, the overweight BMI is a favorable factor of survival in nocturic outpatients without comorbidity or with relatively mild one. This paradox can be compatible to the reported relationship between mortality and BMI after fracture [23].

There are several study limitations in this study. The purpose of this study was to assess the association of BMI and all-cause mortality, while further detailed study into the influence of BMI on the cause-specific mortality would be variable. The association between nocturia and falls/fractures, and its relation to mortality was reported [6, 24–26]. However, the low incidence of death in the present was not enough to evaluate it and we cannot find any case that had a direct relation to fractures and death in the present study except the case of traffic accident. The low incidence of death might not have sufficient power to evaluate the factor of death in nocturic patients, which indicate that the other covariates examined might have a relation to death. Age or sex-related disease could be associated with weight loss and death [17, 27]. In addition, it cannot be denied that other undetected factors are related to the mortality risk increased in low-BMI nocturic patients. Only one series of measures was obtained from participants, even though the survival rates was referred with that of the Japanese population calculated using age- and sex-specific population estimates released by the Japanese Ministry of Internal Affairs and Communications. This design makes it difficult to ascribe directionality to any other observed effects. Other limitations of this study are that participants were all Japanese, so the relationship of the BMI and mortality might be different in other countries. The lowest risk of mortality was observed in the middle and elderly Japanese people with BMI between 21 and 27 [27]. Although the WHO expert consultation agreed the WHO BMI cut-off points retained as international classifications, but the Asians generally have a higher percentage of body fat than Caucasians of the same age, sex, and BMI [28]. Further study is warranted in more ethnically diverse populations. Taking into account these limitations, we believe that our results give novel insights into risk factors of mortality in nocturic patients.

## Conclusion

Additional attention should be paid to nocturic patients with underweight BMI visiting large general hospitals because they may have high mortality. Large prospective studies are needed to validate our findings and extend more.

## Additional file

**Additional file 1.** Charlson Comorbidity Score, Kaplan–Meier of all-cause mortality and analyses predicting death in nocturic patients with Charlson Comorbidity Score 0-2.

## Abbreviations

BMI: body mass index
IPSS: International Prostate Symptom Score
LUTS: lower urinary tract symptoms
SF-36: Medical Outcome Study Short Form-36 item
PCS: Physical Component Summary
MCS: Mental Component Summary
RCS: Role Component Summary
PSQI: Pittsburgh Sleep Quality Index
NPi: Nocturnal Polyuria index
