# Supplementary material for: Underweight body mass index is a risk factor of mortality in outpatients with nocturia in Japan
Source: BMC Res Notes. 2015 Sep 29;8:490. doi: 10.1186/s13104-015-1456-6 (PMC4587784; doi:10.1186/s13104-015-1456-6)
Supplement: Supplementary file 1 — Additional file 1. Charlson Comorbidity Score, Kaplan–Meier of all-cause mortality and analyses predicting death in nocturic patients with Charlson Comorbidity Score 0-2. [file 13104_2015_1456_MOESM1_ESM.pdf]

Supplementary Info.1 Charlson Comorbidity Score.

Scoring: Comorbidity Component (Apply 1 point to each unless otherwise noted)
A. Myocardial Infarction
B. Congestive Heart Failure
C. Peripheral Vascular Disease
D. Cerebrovascular Disease
E. Dementia
F. COPD
G. Connective Tissue Disease
H. Peptic Ulcer Disease
I. Diabetes Mellitus (1 point uncomplicated, 2 points if end-organ damage)
J. Moderate to Severe Chronic Kidney Disease (2 points)
K. Hemiplegia (2 points)
L. Leukemia (2 points)
M. Malignant Lymphoma (2 points)
N. Solid Tumor (2 points, 6 points if metastatic)
O. Liver Disease (1 point mild, 3 points if moderate to severe)

Supplementary Info.2 Kaplan–Meier estimates of all-cause mortality in outpatients with nocturia.

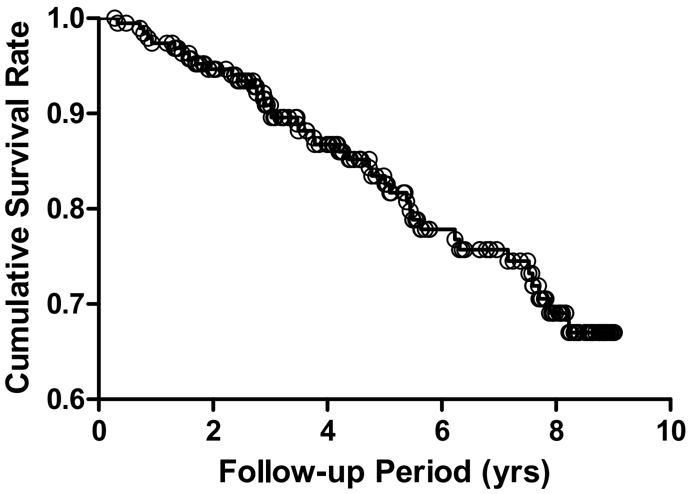

Supplementary Info.3 Characteristics, Cumulative Survival Rate and Univariate/Multivariate cox regression analysis predicting death in nocturic patients with Charlson Comorbidity Score 0-2

Clinical characteristics

Characteristics	BMI			*P* Value*
	<18.50	18.50-24.99	≥25.00	
No. patients	18	112	36	
Age, y, median (IQR)	75 (10)	74 (11)	68 (9)	<.05
Female, No. (%)	7 (38.9)	22 (19.7)	7 (19.4)	.17
BMI, median (IQR)	17.3 (1.8)	21.8 (2.8)	27.0 (3.4)	NA
IPSS score 7, median (IQR)	3.5 (1.3)	4 (1.0)	4 (1)	.80
IPSS total score, median(IQR)	11 (12.3)	15 (8)	15 (10)	.77
IPSS QOL, median (IQR)	5 (1)	5 (1)	4 (1)	.27
SF-36 PCS, median (IQR)	40.3 (22.9)	46.0 (14.7)	47.5 (11.5)	.65
SF-36 MCS, median (IQR)	48.0 (9.1)	50.4 (13.4)	49.2 (13.2)	.46
SF-36 RCS, median (IQR)	38.3 (29.7)	47.1 (22.4)	47.7 (18.1)	.45
PSQI, median (IQR)	9 (5.3)	8 (7)	8 (5.5)	.82
NPi, median (IQR)	0.49 (0.39)	0.43(0.31)	0.45(0.34)	.31
Charlson Comorbidity Score	1 (2)	0 (2)	0 (1)	.21
Follow up, y, median (IQR)	4.47 (3.47)	4.73 (4.72)	7.20 (5.50)	.22

	Univariate Analysis	
	HR (95% CI)	*P* Value*
Age at first visit	1.12 (1.05-1.19)	<.001
Female	0.75 (0.28-1.98)	.75
BMI	0.73 (0.64-0.84)	<.0001
IPSS score 7	1.33 (0.87-2.02)	.18
IPSS total score	1.02 (0.96-1.08)	.52
IPSS QOL	1.13 (0.81-1.59)	.47
SF-36 PCS	0.96 (0.93-0.99)	<.01
SF-36 MCS	0.99 (0.95-1.04)	.67
SF-36 RCS	0.99 (0.96-1.02)	.48
PSQI	1.00 (0.89-1.12)	.93
NPi	1.12 (0.08-16.4)	.93
Charlson Comorbidity Score	1.18 (0.77-1.78)	.45

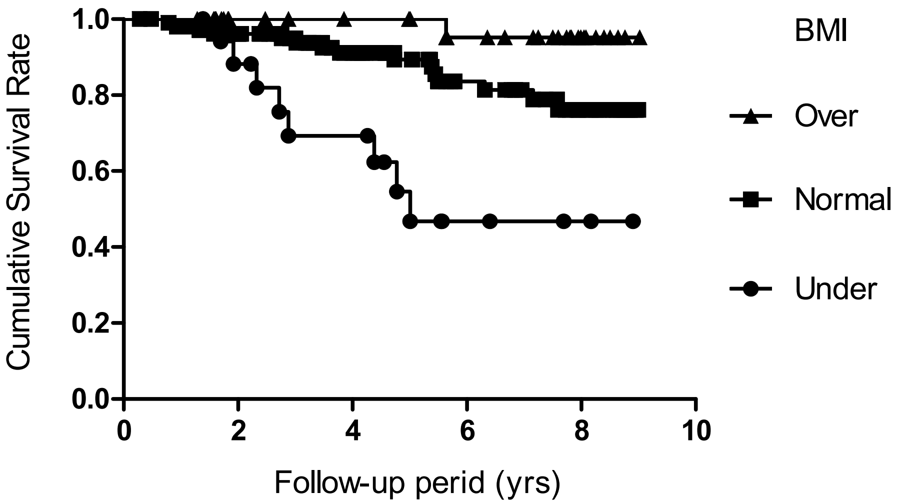

	Multivariate Analysis	
	HR (95% CI)	*P* Value*
Age at first visit	1.08 (1.00-1.17)	.051
BMI	0.73 (0.62-0.88)	<.001
SF-36 PCS	0.98 (0.94-1.01)	.15
